# Chronic Conditions and Multimorbidity Among Middle-Aged and Elderly Peri-Urban Dwellers in Dar es Salaam, Tanzania

**DOI:** 10.3389/ijph.2024.1606387

**Published:** 2024-06-26

**Authors:** Stefan Kohler, Till Bärnighausen, Patrick Kazonda, Germana H. Leyna, Julia Lohmann, Japhet Killewo, Julia K. Rohr, Laura-Marie Stieglitz, Nicolas Paul

**Affiliations:** ^1^ Heidelberg Institute of Global Health, Faculty of Medicine and University Hospital, Heidelberg University, Heidelberg, Germany; ^2^ Institute of Social Medicine, Epidemiology and Health Economics, Charité – Universitatsmedizin Berlin, Corporate Member of Freie Universitat Berlin and Humboldt-Universitat zu Berlin, Berlin, Germany; ^3^ Dar es Salaam Urban Cohort Study, Dar es Salaam, Tanzania; ^4^ Department of Epidemiology and Biostatistics, Muhimbili University of Health and Allied Sciences, Dar es Salaam, Tanzania; ^5^ Department of Global Health and Development, London School of Hygiene and Tropical Medicine, London, United Kingdom; ^6^ Harvard Center for Population and Development Studies, Harvard University, Cambridge, MA, United States

**Keywords:** chronic conditions, infectious diseases, middle-aged, multimorbidity, non-communicable diseases, older adults, sub-Saharan Africa, Tanzania

## Abstract

**Objectives:**

Chronic conditions and multimorbidity affect care needs and prevention opportunities.

**Methods:**

We studied 2,246 men and women aged ≥40 years within the Dar es Salaam Urban Cohort Study from June 2017 to July 2018. Seventeen chronic conditions were assessed based on self-report, body and blood pressure measurement, blood tests, and screening instruments.

**Results:**

Hypertension (51.3%), anemia (34.1%), obesity (32.2%), diabetes (31.6%), depressive symptoms (31.5%), low grip strength (21.2%), and ischemic heart disease (11.9%) were widespread. Multimorbidity was common (73.7%). Women had higher odds of obesity, ischemic heart disease, and high cholesterol (adjusted OR: 2.08–4.16) and lower odds of underweight, low grip strength, alcohol problems, and smoking (adjusted OR: 0.04–0.45). Ten years of age were associated with higher odds of low grip strength, cognitive problems, hypertension, kidney disease, chronic cough, diabetes, high cholesterol, ischemic heart disease, and multimorbidity (adjusted OR: 1.21–1.81) and lower odds of HIV infection (adjusted OR: 0.51).

**Conclusion:**

We found a higher prevalence of multimorbidity than previously estimated for middle-aged and elderly people in sub-Saharan Africa. The chronic conditions underlying multimorbidity differed by sex.

## Introduction

Sub-Saharan Africa has been experiencing an epidemiological transition with a shift in the disease burden from infectious diseases to non-communicable diseases [1–3]. Health systems in this region face the challenge to care for rising levels of non-communicable diseases, still high levels of infectious diseases, and combinations thereof [1, 2]. Aggravating this health system challenge, the population aged ≥65 years in Africa has been predicted to grow from 48 million in 2021 to 569 million in 2100 [4, 5].

In a meta-analysis of 39 studies on low-income and middle-income countries, including 15 studies of five different datasets from sub-Saharan African countries, the estimated pooled prevalence of multimorbidity of non-communicable diseases was 36.4% (95% CI: 32.2–40.6) with a large variation from 0.7% to 81.3% between studies. The prevalence of multimorbidity of non-communicable diseases tended to be higher for the aged, women, people who were well-off, and urban dwellers [6]. Another meta-analysis of multimorbidity of communicable and non-communicable conditions estimated a pooled prevalence of 37.2% (95% CI: 34.9–39.4) globally based on 126 studies from 54 countries and 28.2% (95% CI: 15.6–40.8) for sub-Saharan Africa based on 10 studies [7]. The study conjectured that the low pooled prevalence estimated for sub-Saharan Africa could result from high levels of undiagnosed chronic illness [7].

Previous studies assessing the prevalence of multimorbidity in sub-Saharan Africa were often conducted in South Africa [6–10]. South Africa is one of the few upper middle income countries in the region and maintained an average upper middle income level since 2004 [11]. Studies in Tanzania reported a prevalence of multimorbidity of 25.3% [12], 61.1% [13], and 73.8% (95% CI: 71.2–76.3) for women only [14] among ≥40-year-olds assessing eight, 13, and 15 chronic conditions, respectively, in the same population as this study. A study among ≥60-year-olds in rural Tanzania found a multimorbidity prevalence of 26.1% (95% CI: 16.7–35.4) based on self-report of chronic conditions and 67.3% (95% CI: 57.0–77.5) based on clinical assessment, screening tools, and blood pressure measurement [15]. Another study in Tanzania and Uganda reported a multimorbidity prevalence of 25.6% among people living with HIV, diabetes, and/or hypertension [16]. None of these previous studies of multimorbidity in sub-Sahara Africa reported the prevalence of multimorbidity and underlying conditions by sex and age.

In this study, we evaluated 17 communicable and non-communicable health conditions among ≥40-year-old men and women living in two peri-urban wards of Dar es Salaam, Tanzania. First, we estimated the prevalence of chronic conditions and multimorbidity. Second, we assessed the relationships of chronic conditions and multimorbidity with sex and age.

## Methods

### Study Design and Setting

Tanzania transitioned from a low-income country to a lower middle-income country in 2019 based on the estimated gross national income *per capita* [11]. Dar es Salaam is Tanzania’s largest city and among its wealthier regions. We conducted a cross-sectional study between June 2017 and July 2018 among the peri-urban dwellers living in the Ukonga and Gongolamboto wards of Dar es Salaam. The neighboring Ukonga and Gongolamboto wards are located about 20 km from the city center, were densely populated, and had a mixture of well-built houses and mud houses. Most households had a toilet and about half had electricity [17]. The study was part of the “Health and Aging in Africa: Longitudinal Studies in Three INDEPTH Communities” (HAALSI) project [18].

### Study Population

We randomly selected a sex-stratified sample of 4,850 individuals (2,450 men and 2,400 women) aged ≥40 years from the 2016 Dar es Salaam Health and Demographic Surveillance System, which is also known as the Dar es Salaam Urban Cohort Study (DUCS) [17]. Half of the study sample was randomly selected for additional point-of-care blood tests. Of the individuals selected for the study, 2,299 (744 men and 1,555 women) agreed to participate in a survey and non-invasive health measurement; 1,024 (318 men and 706 women) agreed to undergo additional blood testing. Reasons for non-participation in the survey and non-invasive measurements included unsuccessful attempts to reach a selected participant at home and lack of time to participate. Reasons for not agreeing to blood testing included concerns about confidentiality, the invasiveness of the requested procedures, and religious reasons.

After removing records with missing socioeconomic characteristics, the final study sample comprised up to 2,246 participants for the survey and non-invasive measurements and up to 1,014 participants for blood testing. The analyzed study sample underrepresented men (718 of 2,246; 32.0%) in comparison to the 2016 DUCS population aged ≥40 years (9,067 of 16,898, 54.7%; *p* < 0.001). In the sex-stratified study sample, the share of men aged 40–54 years was lower and the share of men aged ≥55 years was higher than in the 2016 DUCS population (*p* < 0.001). The shares of women aged 40–44 years, 50–54 years, or 70–74 years were lower and the shares of women aged 45–49 years or 55–70 years were higher in the study sample than in the 2016 DUCS population (*p* = 0.002) (Supplementary Table S1; Supplementary Figures S1, S2).

### Data Collection

Field researchers visited study participants at home to conduct a computer-assisted personal interview, take body measurements, and measure blood pressure. The personal interviews included adapted versions of pre-existing screening instruments for chronic conditions. Body measurements included height, weight, and grip strength of the dominant hand. A finger-prick blood sample was taken from a subgroup of the study participants and point-of-care blood tests were performed. The CareSens Blood Glucose Monitoring System and the HemoCue Hemoglobin 201+ Analyser were used for point-of-care blood testing.

### Assessment of Chronic Conditions

#### Self-Report of Chronic Conditions

Ischemic heart disease, hypercholesterolemia, stroke, smoking status, chronic cough (no tuberculosis [TB]), kidney disease, HIV, and tuberculosis were identified by self-reporting either a diagnosis or ever receiving treatment for the respective condition. Diabetes and hypertension were identified either by a self-report of current treatment or by measurement (Supplementary Table S2).

#### Measurement of Chronic Conditions

Anemia, diabetes, hypertension, obesity, and underweight were defined based on thresholds for hemoglobin (<12 mg/dL for women and <13 mg/dL for men), blood glucose (≥200 mg/dL at any time, fasting blood glucose ≥126 mg/dL, or on diabetes treatment), blood pressure (systolic pressure ≥140 mmHg, diastolic pressure ≥90 mmHg, or on antihypertensive treatment), and the body mass index (>30 kg/m^2^ and <18.5 kg/m^2^), respectively. The hemoglobin threshold was adjusted for smoking (+0.3 mg/dL) and African origin (−1 mg/dL) [19, 20]. Hand grip strength <27 kg for men and <16 kg for women was categorized as low and indicative of sarcopenia [21] (Supplementary Table S2).

#### Screening of Chronic Conditions

Ischemic heart disease included a self-reported past diagnosis or screening positive for symptoms of angina pectoris, which were assessed using a modified Rose Angina Questionnaire [22, 23]. Depressive symptoms were assessed using the 10-item Center of Epidemiologic Studies Depression Scale (cut-off score ≥10) [24, 25]. Signs of alcoholism were assessed using the Cut, Annoyed, Guilty, and Eye (CAGE) questionnaire (cut-off score ≥2) [26]. Signs of cognitive problems were assessed using recall tests (cut-off score ≤1.5 SD of sample mean), which we adapted from the United States Health and Retirement Study [27, 28], or self-rated memory (rated as fair or poor) if recall tests were incomplete (Supplementary Table S2).

#### Multimorbidity

Multimorbidity was defined as being affected by ≥2 chronic conditions. As anemia and diabetes were only assessed in a subsample of study participants, we assessed multimorbidity based on either 17 or 15 chronic conditions. The multimorbidity measure based on 17 chronic conditions was the primary multimorbidity outcome assessed. It was only available in the subsample of study participants selected for blood testing. The multimorbidity measure based on 15 chronic conditions excluded anemia and diabetes and was thus available for the full study sample. Both multimorbidity measures were treated as missing data when data on one or more chronic conditions was missing.

### Data Analysis

Characteristics of the study participants were summarized using descriptive statistics. We report the median and interquartile range (IQR) for continuous variables and the number and percentage of observations in a category for categorical variables. Statistical differences between male and female study participants were assessed using the Wilcoxon rank-sum test for continuous variables and the Pearson’s χ^2^ test for categorical variables. The sample prevalence for each chronic condition and multimorbidity was estimated as a proportion with logit-transformed confidence intervals. Tests on the equality of proportions used large-sample statistics. Tetrachoric correlation was used to analyze pairwise correlations between chronic conditions and multimorbidity. For each sex, the relationships of chronic conditions and multimorbidity with age were assessed using point biserial correlation and Epanechnikov kernel-weighted local polynomial regression graphs. The relationships of chronic conditions and multimorbidity with sex and age were further assessed using univariable and multivariable logistic regressions. The multivariable regressions included sex and age together and adjusted for religion, marital status, number of children, literacy, formal education, work status, availability of food, and the location of the dwelling. We rescaled age to 10-year increments to better reflect longer term developments in the regression results. Odds ratios of age (ORs) therefore express changes per 10 years. Standard errors were estimated using the Huber-White sandwich estimator. Statistical significance was assumed for *p* < 0.05. All analyses were conducted in Stata/SE 18.5.

### Ethical Considerations

The institutional review boards of the Muhimbili University of Health and Allied Sciences in Tanzania (2015-04-22/AEC/Vol.IX/82) and the Harvard T.H. Chan School of Public Health in the United States (14-4282) approved the study. Participants gave written informed consent to participate in the study before interviews and, where applicable, again before blood collection and testing.

## Results

### Sample Characteristics

Our study sample of 2,246 participants aged ≥40 years included 32.0% men and 68.0% women. About half of the study participants (48.3%) were 40–49 years old and about one-quarter (27.0%) were 50–59 years old. About one-quarter (24.7%) had reached or surpassed the retirement age of 60 years. Almost all study participants (98.9%) were of Tanzanian origin. About half (54.4%) stated to be Muslim and about half (45.6%) to be Christian. Being married or cohabitant (70.8%) and having children (97.1%) was common. Most study participants could read and/or write (83.8%) and attended less than 7 years or no formal education (77.6%). Almost half (48.8%) of the study participants experienced food insecurity at home due to a lack of money at least once in the past year (Table 1).

**TABLE 1:** Sociodemographic characteristics and health status of ≥40-year-old study participants (Dar es Salaam, Tanzania, 2024).

	Total	Male	Female	*p*
	N ≤ 2,246	N ≤ 718	N ≤ 1,528	
Socioeconomic characteristics				
Age (years)	50 (44–59)	54 (46–63)	49 (43–57)	<0.001
Age group (40–49 years)	1,084 (48.3)	276 (38.4)	808 (52.9)	<0.001
50–59 years	607 (27.0)	199 (27.7)	408 (26.7)	
60–69 years	368 (16.4)	157 (21.9)	211 (13.8)	
≥70 years	187 (8.3)	86 (12.0)	101 (6.6)	
Country of origin (Tanzania)	2,222 (98.9)	709 (98.7)	1,513 (99.0)	0.56
Other	24 (1.1)	9 (1.3)	15 (1.0)	
Religion (Islam)	1,215 (54.1)	384 (53.5)	831 (54.4)	0.69
Christianity	1,031 (45.9)	334 (46.5)	697 (45.6)	
Marital status (married or cohabitant)	1,591 (70.8)	629 (87.6)	962 (63.0)	<0.001
Widowed	389 (17.3)	42 (5.8)	347 (22.7)	
Never married or separated	266 (11.8)	47 (6.5)	219 (14.3)	
Number of children (none)	65 (2.9)	15 (2.1)	50 (3.3)	0.002
1–2	505 (22.5)	133 (18.5)	372 (24.3)	
≥3	1,676 (74.6)	570 (79.4)	1,106 (72.4)	
Can read and/or write	1882 (83.8)	659 (91.8)	1,223 (80.0)	
Formal education (0–6 years)	1742 (77.6)	487 (67.8)	1,255 (82.1)	<0.001
7–10 years	111 (4.9)	55 (7.7)	56 (3.7)	
≥10 years	393 (17.5)	176 (24.5)	217 (14.2)	
Work status (homemaker)	768 (34.2)	91 (12.7)	677 (44.3)	<0.001
Working	1,041 (46.3)	417 (58.1)	624 (40.8)	
Not working	437 (19.5)	210 (29.2)	227 (14.9)	
No food in house, past year (never)	1,150 (51.2)	415 (57.8)	735 (48.1)	<0.001
Rarely (once or twice)	637 (28.4)	190 (26.5)	447 (29.3)	
Sometimes (3–10 times)	173 (7.7)	42 (5.8)	131 (8.6)	
Often (>10 times)	286 (12.7)	71 (9.9)	215 (14.1)	
Ward and area (Ukonga, Markaz)	119 (5.3)	30 (4.2)	89 (5.8)	0.48
Ukonga, Mazizini	384 (17.1)	119 (16.6)	265 (17.3)	
Ukonga, Mongolandege	213 (9.5)	69 (9.6)	144 (9.4)	
Ukonga, Mwembe Madafu	528 (23.5)	184 (25.6)	344 (22.5)	
Gongolamboto, Gongolamboto	290 (12.9)	97 (13.5)	193 (12.6)	
Gongolamboto, Guluka Kwalala	216 (9.6)	68 (9.5)	148 (9.7)	
Gongolamboto, Ulongoni	496 (22.1)	151 (21.0)	345 (22.6)	
Health status				
Health today (good or very good), N = 2,244	850 (37.9)	312 (43.5)	538 (35.3)	<0.001
Moderate	1,171 (52.2)	338 (47.1)	833 (54.6)	
Bad or very bad	223 (9.9)	68 (9.5)	155 (10.2)	
Limitations in activities of daily living, N = 2,233	377 (16.9)	92 (12.9)	285 (18.7)	<0.001

N = 2,246 unless noted otherwise. Median (IQR) or n (%).

Women had a median age of 49 years (IQR: 43–57) and were younger than men who had a median age of 54 years (IQR: 46–63; *p* < 0.001). Men were more often than women married or cohabitant, had ≥3 children, any literacy, or attended formal education (all *p* ≤ 0.002). Women were more often than men working as homemaker or otherwise and were affected by food insecurity in the past year (both *p* < 0.001). Similar portions of men (9.5%) and women (10.2%) reported a bad or very bad health status on the study day, but good or very good health was more frequently reported by men (43.5%) than women (35.3%; *p* < 0.001). Fewer men than women reported one or more limitations in activities of daily living (12.9% versus 18.7%; *p* < 0.001).

### Prevalence of Chronic Conditions and Multimorbidity Across Sexes and Age Groups

Among the ≥40-year-old study participants, 93.9% (95% CI: 92.1–95.3) had one or more chronic conditions. The prevalence of multimorbidity was 73.7% (95% CI: 70.7–76.4) when anemia and diabetes were considered. The prevalence of multimorbidity without anemia and diabetes was 58.5% (95% CI: 56.3–60.6). Between half and every 10th study participant screened positive for one or more of the seven most common chronic conditions. These were hypertension (51.3%, 95% CI: 49.2–53.4), anemia (34.1%, 95% CI: 31.2–37.2), obesity (32.2%, 95% CI: 30.3–34.2), diabetes (31.6%, 95% CI: 28.7–34.5), depressive symptoms (31.5%, 95% CI: 29.6–33.5), low grip strength (21.2%, 95% CI: 19.5–23.0), and ischemic heart disease (11.9%, 95% CI: 10.6–13.3). Signs of alcohol problems were found in 7.7% (95% CI: 6.7–8.9), signs of cognitive problems in 6.6% (95% CI: 5.6–7.7), and HIV infection in 5.1% (95% CI: 4.2–6.1) of the study participants. The seven least prevalent chronic conditions, which affected every 20th or fewer study participants, were high cholesterol (5.0%, 95% CI: 4.2–6.0), tuberculosis (4.8%, 95% CI: 4.0–5.8), stroke (4.8%, 95% CI: 4.0–5.8), current smoking (4.5%, 95% CI: 3.7–5.4), underweight (4.3%, 95% CI: 3.5–5.2), chronic cough (3.3%, 95% CI: 2.6–4.1), and kidney disease (2.9%, 95% CI: 2.3–3.6) (Table 2; Supplementary Table S3).

**TABLE 2:** Prevalence of chronic conditions and multimorbidity among ≥40-year-old study participants (Dar es Salaam, Tanzania, 2024).

Chronic condition	All	Men	Women	*p*	40–59 years	≥60 years	*p*
Hypertension, N = 2,184	51.3 (49.2–53.4)	54.7 (51.0–58.4)	49.7 (47.1–52.2)	0.027	45.2 (42.8–47.6)	69.9 (65.9–73.6)	<0.001
Anemia, N = 984	34.1 (31.2–37.2)	29.1 (24.2–34.5)	36.4 (32.8–40.0)	0.027	33.6 (30.3–37.1)	35.7 (29.9–42.0)	0.562
Obesity, N = 2,141	32.2 (30.3–34.2)	16.1 (13.5–19.0)	39.8 (37.3–42.4)	<0.001	34.3 (32.0–36.6)	25.8 (22.2–29.7)	<0.001
Diabetes, N = 985	31.6 (28.7–34.5)	33.8 (28.6–39.4)	30.6 (27.3–34.2)	0.325	29.8 (26.7–33.2)	36.9 (31.0–43.2)	0.040
Depressive symptoms, N = 2,220	31.5 (29.6–33.5)	29.6 (26.4–33.1)	32.4 (30.1–34.8)	0.192	30.3 (28.1–32.5)	35.5 (31.6–39.7)	0.023
Low grip strength, N = 2,100	21.2 (19.5–23.0)	33.2 (29.8–36.9)	15.6 (13.8–17.5)	<0.001	15.5 (13.8–17.4)	39.2 (35.1–43.6)	<0.001
Ischemic heart disease, N = 2,239	11.9 (10.6–13.3)	6.6 (5.0–8.6)	14.4 (12.8–16.3)	<0.001	10.6 (9.2–12.2)	15.9 (13.1–19.2)	0.001
Signs of alcohol problems, N = 2,234	7.7 (6.7–8.9)	13.0 (10.7–15.7)	5.3 (4.2–6.5)	<0.001	8.1 (6.9–9.5)	6.8 (4.9–9.2)	0.324
Signs of cognitive problems, N = 2,243	6.6 (5.6–7.7)	5.6 (4.1–7.5)	7.0 (5.8–8.4)	0.201	3.4 (2.7–4.4)	16.1 (13.2–19.4)	<0.001
HIV, N = 2,228	5.1 (4.2–6.1)	1.8 (1.1–3.1)	6.6 (5.4–7.9)	<0.001	6.1 (5.0–7.3)	2.0 (1.1–3.6)	<0.001
High cholesterol, N = 2,231	5.0 (4.2–6.0)	3.7 (2.5–5.3)	5.7 (4.6–6.9)	0.044	4.7 (3.8–5.8)	6.0 (4.3–8.4)	0.212
Tuberculosis, N = 2,235	4.8 (4.0–5.8)	4.1 (2.8–5.8)	5.2 (4.2–6.4)	0.244	4.9 (4.0–6.1)	4.6 (3.1–6.7)	0.734
Stroke, N = 2,238	4.8 (4.0–5.8)	4.5 (3.2–6.3)	5.0 (4.0–6.2)	0.603	4.2 (3.3–5.2)	6.9 (5.0–9.3)	0.009
Current smoking, N = 2,234	4.5 (3.7–5.4)	12.2 (10.0–14.8)	0.9 (0.5–1.5)	<0.001	4.1 (3.2–5.1)	5.7 (4.0–8.0)	0.121
Underweight, N = 2,141	4.3 (3.5–5.2)	5.4 (3.9–7.4)	3.7 (2.9–4.8)	0.070	3.6 (2.8–4.7)	6.2 (4.4–8.6)	0.012
Chronic cough (no TB), N = 2,233	3.3 (2.6–4.1)	2.5 (1.6–4.0)	3.6 (2.8–4.7)	0.175	2.7 (2.1–3.6)	4.9 (3.4–7.1)	0.012
Kidney disease, N = 2,231	2.9 (2.3–3.6)	2.1 (1.3–3.5)	3.2 (2.4–4.2)	0.140	2.4 (1.7–3.2)	4.4 (3.0–6.5)	0.014
Multimorbidity (17), N = 915	73.7 (70.7–76.4)	72.2 (66.6–77.3)	74.3 (70.7–77.5)	0.523	70.7 (67.3–74.0)	83.7 (78.0–88.1)	<0.001
Multimorbidity (15), N = 2027	58.5 (56.3–60.6)	56.3 (52.5–60.1)	59.5 (56.9–62.1)	0.178	53.5 (51.0–56.0)	74.9 (70.8–78.6)	<0.001
N	915–2,243	270–717	645–1,526		707–1,689	208–554	

% (#–#) = prevalence (95% logit-transformed confidence intervals), (#) = number of chronic conditions used to assess multimorbidity, TB, tuberculosis. *p*-value for test of equality of proportion between groups.

### Prevalence of Chronic Conditions and Multimorbidity by Sex and Age

**FIGURE 1:**
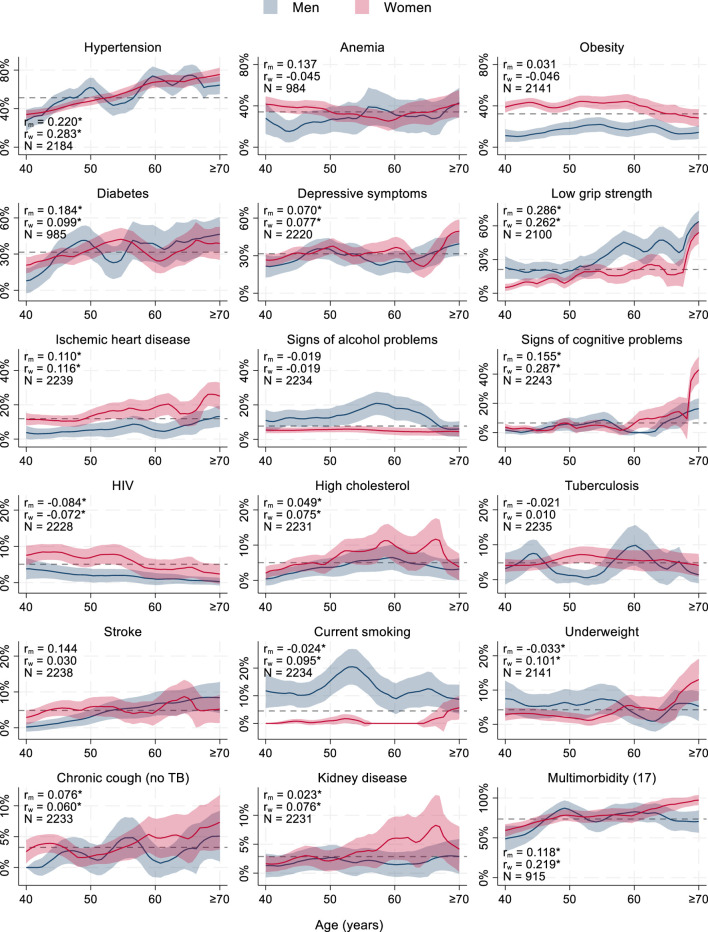
Prevalence of chronic conditions and multimorbidity among ≥40-year-old study participants (Dar es Salaam, Tanzania, 2024) *p < 0.05. r = point biserial correlation coefficient of chronic condition and age, m = men, w = women, N = number of observations, dashed line = average prevalence across ages and sexes (#) = number of chronic conditions used to assess multimorbidity, TB = tuberculosis. Epanechnikov kernel-weighted local polynomial regression with 95% confidence intervals. Scale of *y*-axis differs across graphs.

The prevalence of multimorbidity was similar among men and women irrespectively of whether multimorbidity was assessed based on 17 or 15 chronic conditions. However, the prevalence of the underlying chronic conditions differed between men and women. Hypertension, low grip strength, signs of alcohol problems, and current smoking were more prevalent among men. More prevalent among women were anemia, obesity, signs of ischemic heart disease, HIV, and high cholesterol (all *p* ≤ 0.044). The prevalence of multimorbidity was higher among the study participants of or over the retirement age of 60 years (70.7%, 95% CI: 67.3–74.0 versus 83.7%, 95% CI: 78.0–88.1; *p* < 0.001 with 17 chronic conditions and 53.5%, 95% CI: 51.0–56.0 versus 74.9%, 95% CI: 70.8–78.6; *p* < 0.001 with 15 chronic conditions). Across sexes, this increase was driven by a higher prevalence of hypertension, diabetes, depressive symptoms, low grip strength, ischemic heart disease, signs of cognitive problems, stroke, underweight, chronic cough, and kidney disease at older age (*p* ≤ 0.040). Only obesity and HIV were less prevalent in the ≥60-year-olds compared to younger participants (*p* < 0.001) (Table 2; Figure 1; Supplementary Figures S3, S4).

### Correlation Between Chronic Conditions and Multimorbidity

Pairwise correlations between chronic conditions were often insignificant or low (Tetrachoric correlation r < 0.30). Correlations between chronic conditions and multimorbidity were mostly moderate (r = 0.30–0.50) or strong (r ≥ 0.50). The strongest positive correlations between individual chronic conditions were between signs of alcohol problems and current smoking (r = 0.42), depressive symptoms and ischemic heart disease (r = 0.41), high cholesterol and kidney disease (r = 0.39), obesity and high cholesterol (r = 0.38), HIV and tuberculosis (r = 0.35), current smoking and kidney disease (r = 0.35), hypertension and high cholesterol (r = 0.32), stroke and kidney disease (r = 0.32), tuberculosis and underweight (r = 0.31), signs of alcohol problems and chronic cough (r = 0.30), and smoking and underweight (r = 0.30). The strongest positive correlations (r ≥ 0.50) between individual chronic condition and multimorbidity (based on 17 chronic conditions) occurred for high cholesterol, chronic cough, and kidney disease (all r = 1), ischemic heart disease (r = 0.69), hypertension (r = 0.60), obesity, depressive symptoms (both r = 0.57), and diabetes (r = 0.50) (Table 3).

**TABLE 3:** Pairwise correlations between chronic conditions and multimorbidity among ≥40-year-old study participants (Dar es Salaam, Tanzania, 2024).

	Hypertension	Anemia	Obesity	Diabetes	Depressive symptoms	Low grip strength	Ischemic heart disease	Signs of alcohol problems	Signs of cognitive problems	HIV	High cholesterol	Tuberculosis	Stroke	Current smoking	Underweight	Chronic cough (no TB)	Kidney disease	Multimorbidity (17)	Multimorbidity (15)
Hypertension	1	−0.17*	0.22*				0.17*				0.32*							0.6*	0.64*
Anemia	−0.17*	1	−0.15*			0.14*				0.25*			0.19*					0.49*	
Obesity	0.22*	−0.15*	1			−0.29*	0.22*				0.38*	−0.24*		−0.35*	−1*	0.24*		0.57*	0.62*
Diabetes	0.06	0.002	0.08	1														0.50*	
Depressive symptoms	0.02	0.09	0.06	−0.02	1	−0.14*	0.41*					0.26*						0.57*	0.57*
Low grip strength	0.05	0.14*	−0.29*	0.11	−0.14*	1			0.28*									0.34*	0.36*
Ischemic heart disease	0.17*	0.14	0.22*	0.04	0.41*	0.06	1		0.27*		0.24*	0.26*	0.22*				0.27*	0.69*	0.69*
Signs of alcohol problems	0.05	0.03	0.09	0.04	−0.11	0.11	0.01	1						0.42*		0.30*		0.46*	0.51*
Signs of cognitive problems	0.07	0.15	−0.07	0.14	0.16	0.28*	0.27*	−0.09	1									0.39*	0.48*
HIV	−0.14	0.25*	−0.09	0.03	0.07	−0.003	0.01	0.15	0.01	1		0.35*						0.47*	0.45*
High cholesterol	0.32*	−0.15	0.38*	0.09	−0.06	−0.09	0.24*	−0.03	−1	0.20	1						0.39*	1*	0.63*
Tuberculosis	−0.05	0.12	−0.24*	−0.17	0.26*	−0.0007	0.26*	−0.27	0.22	0.35*	0.03	1			0.31*			0.44*	0.59*
Stroke	0.06	0.19*	−0.13	0.16	−0.01	0.15	0.22*	0.16	0.06	0.15	0.14	−0.14	1				0.32*	0.42*	0.38*
Current smoking	0.06	0.14	−0.35*	0.03	−0.07	0.07	−0.08	0.42*	−0.06	−0.0008	−1	0.13	−1	1	0.30*		0.35*	0.39*	0.43*
Underweight	−0.12	0.20	−1*	−0.18	0.05	0.16	0.03	0.11	0.22	0.21	−1	0.31*	0.08	0.30*	1				0.30*
Chronic cough (no TB)	0.19	−0.11	0.24*	0.19	0.02	0.10	0.02	0.30*	0.28	−1	−0.08	−1	0.13	0	−1	1		1*	1*
Kidney disease	0.16	0.02	−0.04	−0.01	0.18	0.14	0.27*	−0.16	0.16	0.07	0.39*	0.21	0.32*	0.35*	0.02	0.23	1	1*	1*
Multimorbidity (17)	0.60*	0.49*	0.57*	0.50*	0.57*	0.34*	0.69*	0.46*	0.39*	0.47*	1*	0.44*	0.42*	0.39*	0.22	1*	1*	1	1*
Multimorbidity (15)	0.64*		0.62*		0.57*	0.36*	0.69*	0.51*	0.48*	0.45*	0.63*	0.59*	0.38*	0.43*	0.30*	1*	1*	1*	1

N = 915–2,243. *p < 0.05. R = tetrachoric correlation coefficient, (#) = number of chronic conditions used to assess multimorbidity, TB, tuberculosis. Only significant correlations are displayed in the upper right panel.

### Association of Chronic Conditions and Multimorbidity With Sex and Age

In univariable regressions, women had higher odds than men for HIV (OR = 3.77, 95% CI: 2.10–6.77), obesity (OR = 3.46, 95% CI: 2.75–4.35), ischemic heart disease (OR = 2.40, 95% CI: 1.73–3.33), high cholesterol (OR = 1.58, 95% CI: 1.01–2.47), and anemia (OR = 1.39, 95% CI: 1.04–1.87). Women had lower odds than men for current smoking (OR = 0.06, 95% CI: 0.03–0.11), signs of alcohol problems (OR = 0.37, 95% CI: 0.27–0.51), low grip strength (OR = 0.37, 95% CI: 0.30–0.46), and hypertension (OR = 0.82, 95% CI: 0.68–0.98) (Table 4 column 1).

**TABLE 4:** Relationships of chronic conditions with age and sex among ≥40-year-old study participants (Dar es Salaam, Tanzania, 2024).

Chronic condition	Univariable regressions	Univariable regressions	Multivariable regressions	
	Female	Age (10 years)	Female	Age (10 years)
Hypertension, N = 2,184	0.82 (0.68–0.98)*	1.66 (1.51–1.82)*	0.86 (0.69–1.07)	1.52 (1.36–1.70)*
Anemia, N = 984	1.39 (1.04–1.87)*	1.03 (0.91–1.17)	1.35 (0.96–1.90)	0.98 (0.84–1.14)
Obesity, N = 2,141	3.46 (2.75–4.35)*	0.85 (0.78–0.92)*	4.16 (3.23–5.36)*	0.94 (0.85–1.05)
Diabetes, N = 985	0.86 (0.65–1.16)	1.27 (1.12–1.43)*	0.97 (0.69–1.38)	1.25 (1.07–1.46)*
Depressive symptoms, N = 2,220	1.14 (0.94–1.38)	1.17 (1.08–1.27)*	1.15 (0.90–1.46)	1.03 (0.92–1.15)
Low grip strength, N = 2,100	0.37 (0.30–0.46)*	1.99 (1.80–2.20)*	0.33 (0.25–0.44)*	1.81 (1.59–2.05)*
Ischemic heart disease, N = 2,216	2.40 (1.73–3.33)*	1.26 (1.13–1.40)*	3.08 (2.10–4.53)*	1.21 (1.05–1.39)*
Signs of alcohol problems, N = 2,234	0.37 (0.27–0.51)*	0.99 (0.87–1.13)	0.33 (0.23–0.49)*	0.89 (0.74–1.08)
Signs of cognitive problems, N = 2,243	1.28 (0.88–1.86)	2.33 (2.02–2.68)*	0.99 (0.59–1.66)	1.69 (1.40–2.03)*
HIV, N = 2,228	3.77 (2.10–6.77)*	0.62 (0.50–0.76)*	1.89 (0.99–3.61)	0.51 (0.39–0.68)*
High cholesterol, N = 2,208	1.58 (1.01–2.47)*	1.18 (1.05–1.34)*	2.08 (1.26–3.44)*	1.24 (1.01–1.51)*
Tuberculosis, N = 2,235	1.29 (0.84–2.00)	0.95 (0.81–1.11)	1.40 (0.85–2.32)	0.81 (0.65–1.01)
Stroke, N = 2,238	1.12 (0.73–1.71)	1.22 (1.06–1.41)*	1.11 (0.69–1.80)	1.10 (0.91–1.32)
Current smoking, N = 2,234	0.06 (0.03–0.11)*	1.30 (1.11–1.51)*	0.04 (0.02–0.07)*	1.16 (0.94–1.43)
Underweight, N = 2,120	0.67 (0.44–1.04)	1.32 (1.09–1.60)*	0.45 (0.27–0.76)*	1.12 (0.87–1.44)
Chronic cough (no TB), N = 2,233	1.45 (0.84–2.49)	1.32 (1.09–1.59)*	1.48 (0.79–2.79)	1.27 (1.00–1.60)*
Kidney disease, N = 2,166	1.55 (0.86–2.78)	1.26 (1.06–1.51)*	1.87 (0.94–3.73)	1.34 (1.01–1.79)*
Multimorbidity (17), N = 915	1.11 (0.81–1.53)	1.57 (1.31–1.89)*	1.07 (0.74–1.54)	1.39 (1.11–1.75)*
Multimorbidity (15), N = 2027	1.14 (0.94–1.38)	1.71 (1.54–1.90)*	1.26 (1.00–1.58)*	1.54 (1.35–1.75)*
Pseudo *R* ^2^	0.0003–0.169	0.000008–0.148	0.031–0.250	

*p < 0.05. OR (#–#) = odds ratio (95% confidence interval), (#) = number of chronic conditions used to assess multimorbidity, TB, tuberculosis. The multivariable regressions included sex and age and adjusted for religion, marital status, number of children, literacy, formal education, work status, availability of food, and the area of the dwelling.

Older age (per 10 years) was associated with higher odds of signs of cognitive problems (OR = 2.33, 95% CI: 2.02–2.68), low grip strength (OR = 1.99, 95% CI: 1.80–2.20), hypertension (OR = 1.66, 95% CI: 1.51–1.82), underweight (OR = 1.32, 95% CI: 1.09–1.60), chronic cough (no TB) (OR = 1.32, 95% CI: 1.09–1.59), current smoking (OR = 1.30, 95% CI: 1.11–1.51), diabetes (OR = 1.27, 95% CI: 1.12–1.43), ischemic heart disease (OR = 1.26, 95% CI: 1.13–1.40), kidney disease (OR = 1.26, 95% CI: 1.06–1.51), stroke (OR = 1.22, 95% CI: 1.06–1.41), high cholesterol (OR = 1.18, 95% CI: 1.05–1.34), depressive symptoms (OR = 1.17, 95% CI: 1.08–1.27), and multimorbidity (OR = 1.57, 95% CI: 1.31–1.89 based on 17 chronic conditions, or OR = 1.71, 95% CI: 1.54–1.90 based on 15 chronic conditions). Older age was associated with lower odds of HIV (OR = 0.62, 95% CI: 0.50–0.76) and obesity (OR = 0.85, 95% CI: 0.78–0.92) (Table 4 column 2).

In multivariable regressions which included sex, age, and other socioeconomic characteristics, women had higher odds than men for obesity (adjusted OR = 4.16, 95% CI: 3.23–5.36), ischemic heart disease (adjusted OR = 3.08, 95% CI: 2.10–4.53), high cholesterol (adjusted OR = 2.08, 95% CI: 1.26–3.44), and multimorbidity based on 15 chronic conditions (adjusted OR = 1.26, 95% CI: 1.00–1.58). Women had lower odds than men for underweight (adjusted OR = 0.45, 95% CI: 0.27–0.76), low grip strength (adjusted OR = 0.33, 95% CI: 0.25–0.44), signs of alcohol problems (adjusted OR = 0.33, 95% CI: 0.23–0.49), and smoking (adjusted OR = 0.04, 95% CI: 0.02–0.07). Older age was associated with higher odds (per 10 years) of low grip strength (adjusted OR = 1.81, 95% CI: 1.59–2.05), signs of cognitive problems (adjusted OR = 1.69, 95% CI: 1.40–2.03), hypertension (adjusted OR = 1.52, 95% CI: 1.36–1.70), kidney disease (adjusted OR = 1.34, 95% CI: 1.01–1.79), chronic cough (no TB) (adjusted OR = 1.27, 95% CI: 1.00–1.60), diabetes (adjusted OR = 1.25, 95% CI: 1.07–1.46), high cholesterol (adjusted OR = 1.24, 95% CI: 1.01–1.51), ischemic heart disease (adjusted OR = 1.21, 95% CI: 1.05–1.39), and multimorbidity (adjusted OR = 1.39, 95% CI: 1.11–1.75 based on 17 chronic conditions and adjusted OR = 1.54, 95% CI: 1.35–1.75 based on 15 chronic conditions). Older age (per 10 years) was associated with lower odds of HIV (adjusted OR = 0.51, 95% CI: 0.39–0.68). Sex ceased to be associated with HIV, anemia, and hypertension after adjusting for age and other socioeconomic characteristics. In turn, women had lower odds of underweight and higher odds of multimorbidity (based on 15 chronic conditions without anemia and diabetes) after adjusting for age and other socioeconomic characteristics. Age ceased to be associated with underweight, smoking, stroke, depressive symptoms, and obesity after adjusting for sex and other socioeconomic characteristics (Table 4 columns 3–4).

## Discussion

### Main Findings of This Study

Multimorbidity based on 17 chronic conditions affected 73.7% of the ≥40-year-old study participants living in the peri-urban Ukonga and Gongolamboto wards of Dar es Salaam. Multimorbidity was associated with age and 83.7% of the ≥60-year-old study participants had multiple chronic conditions. After adjusting for other socioeconomic characteristics, women had higher odds of obesity, ischemic heart disease, and high cholesterol and they had lower odds of underweight, low grip strength, alcohol problems, and smoking. Age was associated with higher adjusted odds of low grip strength, cognitive problems, hypertension, kidney disease chronic cough, diabetes, high cholesterol, ischemic heart disease, and multimorbidity and with lower adjusted odds of HIV infection.

### What Is Already Known

Previous estimates of multimorbidity of non-communicable diseases in low-income countries in sub-Saharan Africa were variable and mostly lower than our estimates [6–9]. A cross-sectional, population-based study in rural and urban Malawi reported that 1%–4% of men and 4%–7% of women had combinations of hypertension, diabetes, and obesity [29]. A cross-country study using the World Health Surveys (WHS) reported a prevalence of multimorbidity of 3.6% in Ghana, 4.2% in Kenya, 6.3% in Burkina Faso, and 7.9% in Namibia [30]. A cross-sectional study among adults in Botswana estimated a multimorbidity prevalence of 5.4% [31]. The Study on global AGEing and adult health (SAGE) reported 22% multimorbidity in a population-based sample from Ghana [32]. A cross-sectional study among community-dwelling people aged ≥60 in Burkina Faso found a multimorbidity prevalence of 65% [33]. Another study reported a 17.8% prevalence of non-communicable chronic diseases among ≥18-year-old hospital patients in Ethiopia [34].

Considering both communicable and non-communicable diseases, multimorbidity was estimated in 28.7% of the people living in two urban slums in Nairobi [35], in 15.3% of adults living with HIV in Zimbabwe [36], in 38.8% of patients of an urban clinic in Ghana [37], in 59.1% of elderly hospital patients in Ethiopia [38], and in 49% of elderly hospital patients in Nigeria [39]. A cross-country study of adults in six urban and rural centers in Ghana, Burkina Faso, Kenya, and South Africa reported a multimorbidity prevalence of 20.2%–51.7% among men and 24.1%–64.9% among women [10]. A previous study in five rural villages in northern Tanzania in 2017 found a prevalence of multimorbidity of 26.1% when chronic conditions were self-reported and 67.3% when chronic conditions were assessed through clinician diagnosis, screening tools, and blood pressure measurement [15]. A study among people living with HIV, diabetes, and/or hypertension in Tanzania or Uganda reported a multimorbidity prevalence of 25.6% [16].

Previous studies of the same sample of ≥40-year-old people in peri-urban Dar es Salaam reported a multimorbidity prevalence of 25.3% [12], 61.1% [13], and 73.8% (95% CI: 71.2–76.3) for women only [14]. Differences in multimorbidity estimates in the same sample result from assessing fewer chronic conditions (8, 13, and 15, respectively) and/or using only self-report [12] in the assessment of chronic conditions. Across studies, older adults were consistently more likely to have multimorbidity [12, 14, 29–37]. Several previous studies further reported that women were more likely multimorbid than man [12, 29–32, 35–37].

### What This Study Adds

Multimorbidity has been called a priority for global health research [40]. A recent systematic review and meta-analysis estimated a pooled multimorbidity prevalence of 28.2% (95% CI: 15.6–40.8) in sub-Saharan Africa and conjectured that this low estimate could indicate high levels of undiagnosed chronic illness in this region [7]. We studied multimorbidity in a ≥40-year-old population based on assessing either 17 or 15 chronic conditions through a combination of self-report, measurement, and screening instruments. We estimated a prevalence of multimorbidity that is higher than previous estimates for sub-Saharan Africa and among the highest estimates that have been reported for low-income and middle-income countries [6]. Our estimate is consistent with a suspected large gap in diagnosing multimorbidity. Even more, finding 70.7% multimorbidity among the 40–59-year-old study participants and 83.7% multimorbidity among the ≥60-year-old study participants suggests that already most middle-aged people living in peri-urban Dar es Salaam and similar settings might benefit from a broad health assessment and subsequent care for multiple health conditions. Our findings further indicate that a broad assessment of chronic conditions can help detect different patterns of multimorbidity, as the chronic conditions determining multimorbidity differ between men and women. For instance, we estimated an association between multimorbidity and being a woman only when multimorbidity was assesses based on 15 chronic conditions without anemia and diabetes.

### Practical Implications

Past studies have identified gaps in the preparedness of health facilities in Tanzania to treat non-communicable diseases such as hypertension and diabetes [41, 42]. A community-based survey from South Africa indicated that 93% of participants who screened positive for diabetes and 58% who screened positive for hypertension had unmet health needs [43]. We found hypertension and diabetes to be the most common and fourth most common chronic conditions, respectively. The study at hand further indicates that multiple chronic conditions are likely to be present among peri-urban dwellers in Tanzania aged ≥40-years. These findings underscore the scale of the challenge to develop care structures for multiple chronic conditions, especially for multiple non-communicable diseases, and broad health assessments for aging people in sub-Saharan Africa.

Approaches to caring for multiple chronic conditions may entail the integration of health programs for single diseases. A study, which piloted integrated HIV, diabetes, and hypertension care in ten health facilities offering primary care in Dar es Salaam and Kampala, has concluded that integrated management of chronic diseases is a feasible strategy [44]. A systematic review and meta-analysis on the effectiveness of integrated chronic care models in sub-Saharan Africa found positive effects on systolic blood pressure and mixed results for other health outcomes [45]. Expanding integrated care programs seems needed globally and continues to require addressing knowledge gaps, for instance, on joint care for communicable and non-communicable diseases [46], the costs of multimorbidity in low- and middle income countries [47], and the components making integrated care for multiple chronic conditions effective and cost-effective in Tanzania and other sub-Saharan African countries.

### Strengths and Limitations

Strengths of this study include, first, that a wide range of chronic conditions was assessed in a general population. Second, we emphasized sensitivity in our assessment of chronic conditions by combining measurement, screening instruments, and self-report of chronic conditions. Third, we assessed multimorbidity and underlying chronic conditions by sex and age. Limitations of this study include, first, that the screening instruments have not been validated in our study population. Second, we used different approaches to assess chronic conditions, namely, self-reporting, screening instruments, and measurement. Seven chronic conditions were based only on self-report, which can be more prone to errors and bias than the use of screening instruments or measurement. Third, we used lifetime measures to assess the presence of chronic conditions. It is possible that certain conditions were no longer present at the time of data collection. Fourth, our study is likely affected by a selection bias, as only 2,299 of the randomly selected 4,840 individuals participated in the study and only 1,024 of 2,420 selected individuals agreed to undergo blood testing. Men were included less often than women in the study. People with few chronic conditions, who worked further away, or with severe comorbidities might have been less likely to participate in the study. We aimed to account for selective participation and non-responses by stratifying descriptive analyses by sex and age and by adjusting for sex, age, and other socioeconomic characteristics in the regression analyses. Fifth, it remains unknown how the prevalence of chronic conditions in the Dar es Salaam Urban Cohort Study (DUCS) population, from which we sampled, compares to other peri-urban or urban populations in Tanzania and elsewhere. Sixth, the study was conducted in 2017/18. The COVID-19 pandemic and other factors could have caused changes in the prevalence of chronic conditions. Seventh, multiple comparisons may have inflated the number of significant findings. Finally, the cross-sectional study design was not suitable to assess causal relationships.

### Conclusion

The comprehensive assessment of chronic conditions and multimorbidity in this study suggests that multiple chronic conditions affect most middle-aged and elderly people in Tanzania. The prevalence of multimorbidity among ≥40-year-old peri-urban dwellers was higher than estimates for sub-Saharan Africa from previous studies. The estimated multimorbidity prevalence of 73.7% suggests a substantial need for the care and prevention of multimorbidity and chronic conditions in Tanzania. Sex and age differences in the prevalence of chronic conditions indicate that women and men have different causes of multimorbidity across the age spectrum that might require different screening, treatment, and preventive care.

## Data Availability

The data and code supporting the findings of this study are openly available in Harvard Dataverse at https://doi.org/10.7910/DVN/9EH23J and heiDATA at https://doi.org/10.11588/data/VVKWZK, respectively.
