# Supplementary material for: Chronic Conditions and Multimorbidity Among Middle-Aged and Elderly Peri-Urban Dwellers in Dar es Salaam, Tanzania
Source: Int J Public Health. 2024 Jun 26;69:1606387. doi: 10.3389/ijph.2024.1606387 (PMC11233465; doi:10.3389/ijph.2024.1606387)
Supplement: Supplementary file 1 [file DataSheet1.docx]

**Supplementary material**

**Table S1:** Age structure of the HAALSI study sample and the Dar Es Salaam Urban Cohort Study (Dar es Salaam, Tanzania, 2016–2020)

(a) Men

|  | **DUCS 2019–20** | **DUCS 2016** | **HAALSI TZ selected** | **HAALSI TZ analyzed^*^** |
| --- | --- | --- | --- | --- |
| **Age group** | | | | |
| 40–44 years | 5090 (31.4) | 3004 (33.1) | 716 (29.2) | 132 (18.4) |
| 45–49 years | 3715 (22.9) | 1972 (21.7) | 559 (22.8) | 144 (20.1) |
| 50–54 years | 2513 (15.5) | 1348 (14.9) | 382 (15.6) | 98 (13.6) |
| 55–59 years | 1628 (10) | 1069 (11.8) | 305 (12.4) | 101 (14.1) |
| 60–64 years | 1246 (7.7) | 717 (7.9) | 190 (7.8) | 84 (11.7) |
| 65–69 years | 860 (5.3) | 467 (5.2) | 154 (6.3) | 73 (10.2) |
| 70–74 years | 521 (3.2) | 242 (2.7) | 72 (2.9) | 41 (5.7) |
| 75+ years | 646 (4) | 248 (2.7) | 72 (2.9) | 45 (6.3) |
| Total | 16219 (100) | 9067 (100) | 2450 (100) | 718 (100) |
|  | | | | |
| **P** | **Comparison** | | | |
| <0.001 |  |  | X | X |
| <0.001 |  | X |  | X |
| <0.001 | X |  |  | X |
| 0.018 |  | X | X |  |
| 0.001 | X |  | X |  |
| <0.001 | X | X |  |  |

(b) Women

|  | **DUCS 2019–20** | **DUCS 2016** | **HAALSI TZ selected** | **HAALSI TZ analyzed^*^** |
| --- | --- | --- | --- | --- |
| **Age group** | | | | |
| 40–44 years | 4965 (34.4) | 2808 (35.9) | 796 (33.2) | 469 (30.7) |
| 45–49 years | 3289 (22.8) | 1545 (19.7) | 525 (21.9) | 339 (22.2) |
| 50–54 years | 1892 (13.1) | 1222 (15.6) | 360 (15) | 237 (15.5) |
| 55–59 years | 1431 (9.9) | 789 (10.1) | 251 (10.5) | 171 (11.2) |
| 60–64 years | 925 (6.4) | 570 (7.3) | 175 (7.3) | 131 (8.6) |
| 65–69 years | 644 (4.5) | 335 (4.3) | 110 (4.6) | 80 (5.2) |
| 70–74 years | 426 (2.9) | 210 (2.7) | 64 (2.7) | 32 (2.1) |
| 75+ years | 880 (6.1) | 352 (4.5) | 119 (5) | 69 (4.5) |
| Total | 14452 (100) | 7831 (100) | 2400 (100) | 1528 (100) |
|  | | | | |
| **P** | **Comparison** | | | |
| 0.443 |  |  | X | X |
| 0.002 |  | X |  | X |
| <0.001 | X |  |  | X |
| 0.194 |  | X | X |  |
| 0.031 | X |  | X |  |
| <0.001 | X | X |  |  |

n (%). ^*^Upper bound. DUCS = Dar Es Salaam Urban Cohort Study, HAALSI = Health and Aging in Africa: Longitudinal Studies in three INDEPTH Communities, TZ = Tanzania. In similar pairwise comparisons of samples and populations, the sex structure differed between HAALSI TZ analyzed, HAALSI TZ selected, DUCS 2016 and DUCS 2019–20 (all P ≤ 0.002) but not between DUCS 2016 and DUCS 2019–20 (P = 0.104).

**Table S2:** Assessment of 17 chronic conditions and health status in the HAALSI study (Dar es Salaam, Tanzania, 2017–2018)

| **Chronic condition** | **Assessment** | **Definition** |
| --- | --- | --- |
| Anemia | 1. Hemoglobin point-of-care test  2. Self-report of smoking | Hemoglobin <12 mg/dl for women and hemoglobin <13 mg/dl for men; threshold adjustments for smoking (+0.3 mg/dl) and African origin (−1 mg/dl) |
| Signs of cognitive problems | Self-rated present memory, immediate and delayed word recall of 10 nouns, date, and president naming; scoring of correct answers; adapted from US Health and Retirement Study [1, 2] | Present memory rated as fair or poor, score ≤ 1.5 standard deviation of population mean |
| Chronic cough | Self-reported coughing (no tuberculosis) | Self-report of usually coughing and not having tuberculosis as a chronic condition |
| Signs of depression | 10-item Centre for Epidemiological Studies Depression Scale (CES-D-10) [3, 4] | CES-D-10 score ≥10 |
| Diabetes | 1. Blood glucose point-of-care test  2. Self-report of current treatment | Blood glucose ≥200 mg/dl, fasting blood glucose ≥126 mg/dl, or currently on treatment for diabetes with diet, weight loss, pills, or insulin injection |
| Kidney disease | Self-reported diagnosis | Ever diagnosed with kidney disease or low kidney function |
| HIV | Self-reported diagnosis | Ever tested HIV positive |
| Hypercholesterolemia | Self-reported diagnosis | Ever diagnosed with high cholesterol |
| Hypertension | 1. Blood pressure was measured according to World Health Organization standards up to three times; the mean of all measurements was calculated  2. Self-report of current treatment | Systolic pressure ≥140 mmHg, diastolic pressure ≥90 mmHg or currently on treatment for hypertension |
| Ischemic heart disease | 1. Self-reported diagnosis of angina, heart failure, or heart attack  2. Modified Rose Angina Questionnaire [5, 6] | Ever received diagnosis of angina, ever received diagnosis of heart attack, or modified Rose Angina Questionnaire criteria |
| Obesity | Measured weight and height | Body mass index >30 kg/m^2^ |
| Signs of sarcopenia | Measured grip strength | Grip strength <27 kg for man, grip strengths <16 kg for women |
| Stroke | 1. Self-reported diagnosis  2. Self-reported treatment | Ever diagnosed with stroke, mini-stroke, transient ischemic attack; suddenly lost half vision, or ever received treatment for stroke |
| Signs of alcoholism | Cut, Annoyed, Guilty, and Eye (CAGE) questionnaire [7] | CAGE score ≥2 |
| Current smoking | Self-reported diagnosis | Self-reported current smoking of any tobacco products, such as cigarettes, cigars, or pipes. |
| Tuberculosis | 1. Self-reported diagnosis  2. Self-reported treatment | Ever diagnosed with tuberculosis, but never received treatment, or currently on tuberculosis treatment |
| Underweight | Measured weight and height | Body mass index <18.5 kg/m^2^ |

Self-reported diagnoses were obtained by asking: Have you ever been told by a doctor, nurse, or other healthcare worker that you have had … ? Modified Rose Angina Questionnaire criteria were, first, experienced any pain or discomfort in the chest or pain going to the left arm or neck when walking uphill or hurrying during the last 12 months; second, either stopping and slowing down or carrying on after taking a pain-relieving medicine that dissolves in the mouth (e.g., a nitro spray or tablet) when getting pain or discomfort while walking; and, third, pain or discomfort is relieved when standing still.

**Table S3:** Prevalence of chronic conditions and multimorbidity among 40–59-year-old and ≥60-year-old study participants (Dar es Salaam, Tanzania, 2024)

| **Chronic condition** | **All** | **Men** | | | **Women** | | | **†** | **#** |
| --- | --- | --- | --- | --- | --- | --- | --- | --- | --- |
|  |  | **40–59 years^†^** | **≥60 years^#^** | **P** | **40–59 years^†^** | **≥60 years^#^** | **P** | **P** | **P** |
| Hypertension, N = 2184 | 51.3 (49.2–53.4) | 51.3 (49.2–53.4) | 68.2 (62.0–73.8) | <0.001 | 44.2 (41.4–47.1) | 71.2 (65.8–76.1) | <0.001 | 0.206 | 0.446 |
| Anemia, N = 984 | 34.1 (31.2–37.2) | 34.1 (31.2–37.2) | 34.6 (26.1–44.2) | 0.119 | 36.3 (32.4–40.4) | 36.6 (28.8–45.1) | 0.954 | 0.010 | 0.749 |
| Obesity, N = 2141 | 32.2 (30.3–34.2) | 32.2 (30.3–34.2) | 16.7 (12.5–22.1) | 0.728 | 41.4 (38.6–44.3) | 33.2 (28.0–38.9) | 0.011 | <0.001 | <0.001 |
| Diabetes, N = 985 | 31.6 (28.7–34.5) | 31.6 (28.7–34.5) | 41.1 (32.1–50.8) | 0.045 | 29.9 (26.2–33.9) | 33.6 (26.0–42.1) | 0.406 | 0.958 | 0.228 |
| Depressive symptoms, N = 2220 | 31.5 (29.6–33.5) | 31.5 (29.6–33.5) | 32.9 (27.2–39.2) | 0.181 | 31.1 (28.6–33.8) | 37.5 (32.2–43.1) | 0.034 | 0.213 | 0.270 |
| Low grip strength, N = 2100 | 21.2 (19.5–23.0) | 21.2 (19.5–23.0) | 48.6 (42.1–55.2) | <0.001 | 11.6 (9.9–13.6) | 31.8 (26.6–37.5) | <0.001 | <0.001 | <0.001 |
| Ischemic heart disease, N = 2239 | 11.9 (10.6–13.3) | 11.9 (10.6–13.3) | 9.1 (6.1–13.5) | 0.049 | 12.7 (10.9–14.7) | 21.2 (17.0–26.1) | <0.001 | <0.001 | <0.001 |
| Signs of alcohol problems, N = 2234 | 7.7 (6.7–8.9) | 7.7 (6.7–8.9) | 10.4 (7.1–15.0) | 0.141 | 5.6 (4.4–7.1) | 3.9 (2.2–6.8) | 0.234 | <0.001 | 0.003 |
| Signs of cognitive problems, N = 2243 | 6.6 (5.6–7.7) | 6.6 (5.6–7.7) | 9.1 (6.0–13.4) | 0.003 | 3.3 (2.4–4.5) | 21.5 (17.3–26.4) | <0.001 | 0.616 | <0.001 |
| HIV, N = 2228 | 5.1 (4.2–6.1) | 5.1 (4.2–6.1) | 0.4 (0.1–2.9) | 0.044 | 7.4 (6.1–9.1) | 3.2 (1.7–5.9) | 0.008 | <0.001 | 0.020 |
| High cholesterol, N = 2231 | 5.0 (4.2–6.0) | 5.0 (4.2–6.0) | 4.2 (2.3–7.6) | 0.594 | 5.2 (4.1–6.6) | 7.5 (5.0–11.0) | 0.124 | 0.115 | 0.110 |
| Tuberculosis, N = 2235 | 4.8 (4.0–5.8) | 4.8 (4.0–5.8) | 4.2 (2.2–7.6) | 0.919 | 5.3 (4.1–6.7) | 4.9 (3.0–7.9) | 0.774 | 0.279 | 0.695 |
| Stroke, N = 2238 | 4.8 (4.0–5.8) | 4.8 (4.0–5.8) | 7.5 (4.7–11.6) | 0.006 | 4.6 (3.6–6.0) | 6.4 (4.2–9.8) | 0.190 | 0.125 | 0.633 |
| Current smoking, N = 2234 | 4.5 (3.7–5.4) | 4.5 (3.7–5.4) | 10.0 (6.8–14.5) | 0.204 | 0.5 (0.2–1.1) | 2.3 (1.1–4.7) | 0.002 | <0.001 | <0.001 |
| Underweight, N = 2141 | 4.3 (3.5–5.2) | 4.3 (3.5–5.2) | 3.9 (2.0–7.3) | 0.201 | 2.6 (1.9–3.7) | 8.1 (5.5–12.0) | <0.001 | 0.001 | 0.046 |
| Chronic cough (no TB), N = 2233 | 3.3 (2.6–4.1) | 3.3 (2.6–4.1) | 3.3 (1.7–6.5) | 0.327 | 3.0 (2.1–4.1) | 6.2 (4.0–9.5) | 0.007 | 0.333 | 0.128 |
| Kidney disease, N = 2231 | 2.9 (2.3–3.6) | 2.9 (2.3–3.6) | 2.1 (0.9–4.9) | 0.985 | 2.5 (1.7–3.5) | 6.2 (4.0–9.5) | 0.001 | 0.660 | 0.021 |
| Multimorbidity (17), N = 915 | 73.7 (70.7–76.4) | 73.7 (70.7–76.4) | 75.5 (65.7–83.3) | 0.375 | 70.8 (66.8–74.5) | 90.4 (83.3–94.6) | <0.001 | 0.928 | 0.004 |
| Multimorbidity (15), N = 2027 | 58.5 (56.3–60.6) | 58.5 (56.3–60.6) | 66.5 (59.8–72.6) | <0.001 | 54.4 (51.4–57.2) | 81.7 (76.5–85.9) | <0.001 | 0.280 | <0.001 |
| N | 915–2243 | 176–475 | 94–242 |  | 531–1214 | 114–312 |  | | |

% (#–#) = prevalence (95% logit-transformed confidence intervals), (#) = number of chronic conditions used to assess multimorbidity, TB = tuberculosis, †/# = comparison of age group over sex. P-value for test of equality of proportion between groups.

**Figure S1:** Flow chart of sample selection in the HAALSI study (Dar es Salaam, Tanzania, 2017–2018)


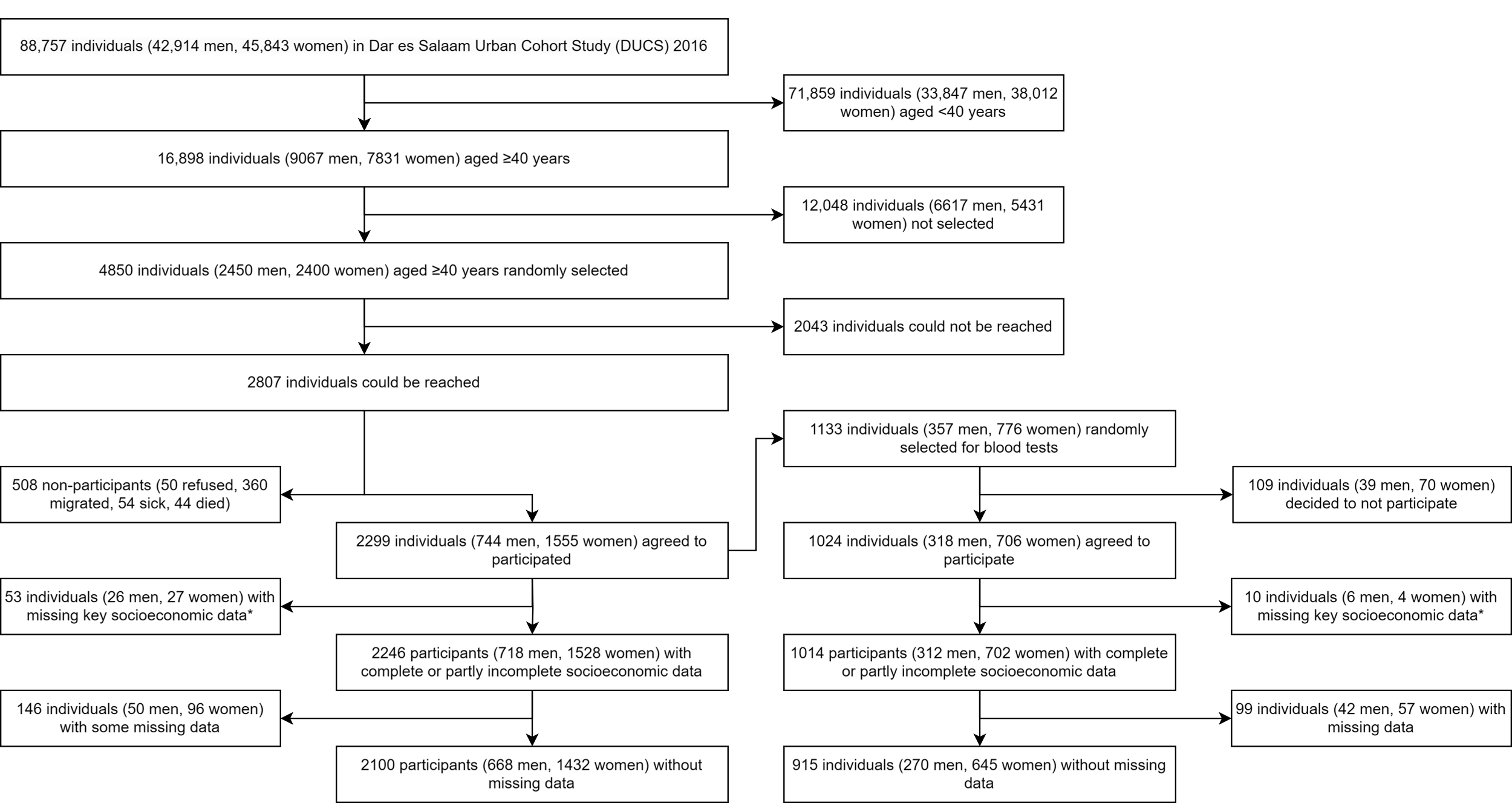


^*^Marital status, number of children, literacy, formal education, work status.

**Figure S2:** Age structure of the HAALSI study sample and the Dar Es Salaam Urban Cohort Study (Dar es Salaam, Tanzania, 2016–2020)

| (a) Men | (b) Women |
| --- | --- |
|  |  |

DUCS = Dar Es Salaam Urban Cohort Study, HAALSI = Health and Aging in Africa: Longitudinal Studies in three INDEPTH Communities. The analyzed HAALSI Tanzania study sample included up to 718 men and 1528 women aged ≥40 years. In the DUCS 2016, 9067 (21.1%) of 42914 men and 7831 (17.1%) of 45843 were aged ≥40 years. In the DUCS 2019–20, 16219 (26.8%) of 60485 of the men and 14452 (21.3%) of 67832 were aged ≥40 years in 2019–20.

**Figure S3:** Prevalence of chronic conditions and multimorbidity among ≥40-year-old study participants by sex and retirement age (Dar es Salaam, Tanzania, 2017–2018)

(a) Men and women (N = 915–2243)

(b) Men (N = 270–717)

(c) Women (N = 645–1526)

**Figure S3 (continued):** Prevalence of chronic conditions and multimorbidity among ≥40-year-old study participants by sex and retirement age (Dar es Salaam, Tanzania, 2017–2018)

(d) 40–59 years (N = 707–1689)

(e) ≥60 years (N = 208–554)

Error bars represent 95% logit-transformed confidence intervals. chronic cough = chronic cough and no tuberculosis, (#) = number of chronic conditions used to assess multimorbidity.

**Figure S4:** Prevalence of multimorbidity based on 17 versus 15 chronic conditions among ≥40-year-old study participants by sex and age (Dar es Salaam, Tanzania, 2017–2018)

^*^P < 0.05. r = point biserial correlation coefficient of chronic condition and age, m = men, w = women, N = number of observations, dashed line = average prevalence across ages and sexes, (#) = number of chronic conditions used to assess multimorbidity. Epanechnikov kernel-weighted local polynomial regression with 95% confidence intervals. Scale of y-axis differs across graphs.

**References**

1. Kobayashi, L.C., et al., *Childhood deprivation and later-life cognitive function in a population-based study of older rural South Africans.* Social Science & Medicine, 2017. **190**: p. 20-28.

2. Kobayashi, L.C., et al., *Cognitive Function and Impairment in Older, Rural South African Adults: Evidence from "Health and Aging in Africa: A Longitudinal Study of an INDEPTH Community in Rural South Africa".* Neuroepidemiology, 2019. **52**(1-2): p. 32-40.

3. Radloff, L.S., *The CES-D Scale: A Self-Report Depression Scale for Research in the General Population.* Applied Psychological Measurement, 1977. **1**(3): p. 385-401.

4. Andresen, E.M., et al., *Screening for depression in well older adults: evaluation of a short form of the CES-D (Center for Epidemiologic Studies Depression Scale).* American Journal of Preventive Medicine, 1994. **10**(2): p. 77-84.

5. Rose, G.A., *The diagnosis of ischaemic heart pain and intermittent claudication in field surveys.* Bulletin of the World Health Organization, 1962. **27**: p. 645-58.

6. Rose, G., P. McCartney, and D.D. Reid, *Self-administration of a questionnaire on chest pain and intermittent claudication.* British Journal of Preventive and Social Medicine, 1977. **31**(1): p. 42-48.

7. Ewing, J.A., *Detecting Alcoholism: The CAGE Questionnaire.* JAMA, 1984. **252**(14): p. 1905-1907.
